# Supplementary figures and images for: An mHealth tool for community health workers to improve caregiver knowledge of child health in the Amazon: An effectiveness-implementation hybrid evaluation
Source: PLOS Glob Public Health. 2022 Sep 22;2(9):e0001118. doi: 10.1371/journal.pgph.0001118 (PMC10021143; doi:10.1371/journal.pgph.0001118)

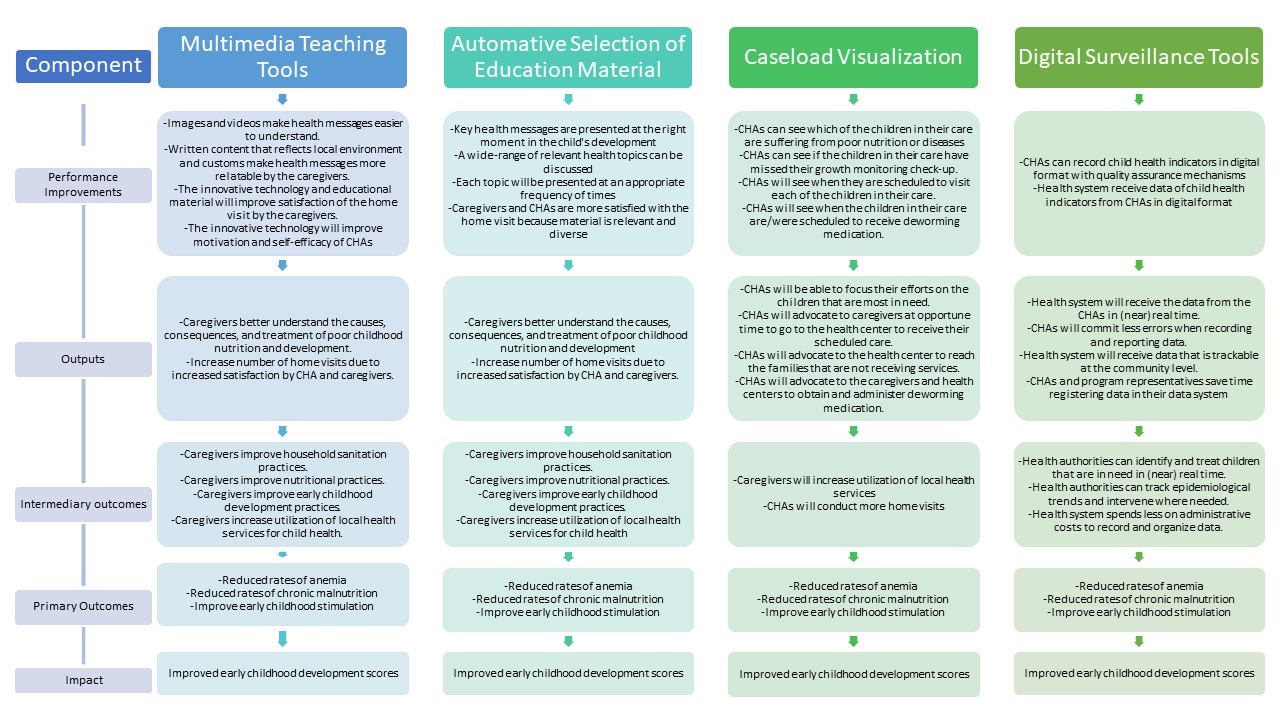

Supplement: S1 Fig — (TIFF) [file pgph.0001118.s001.tiff]

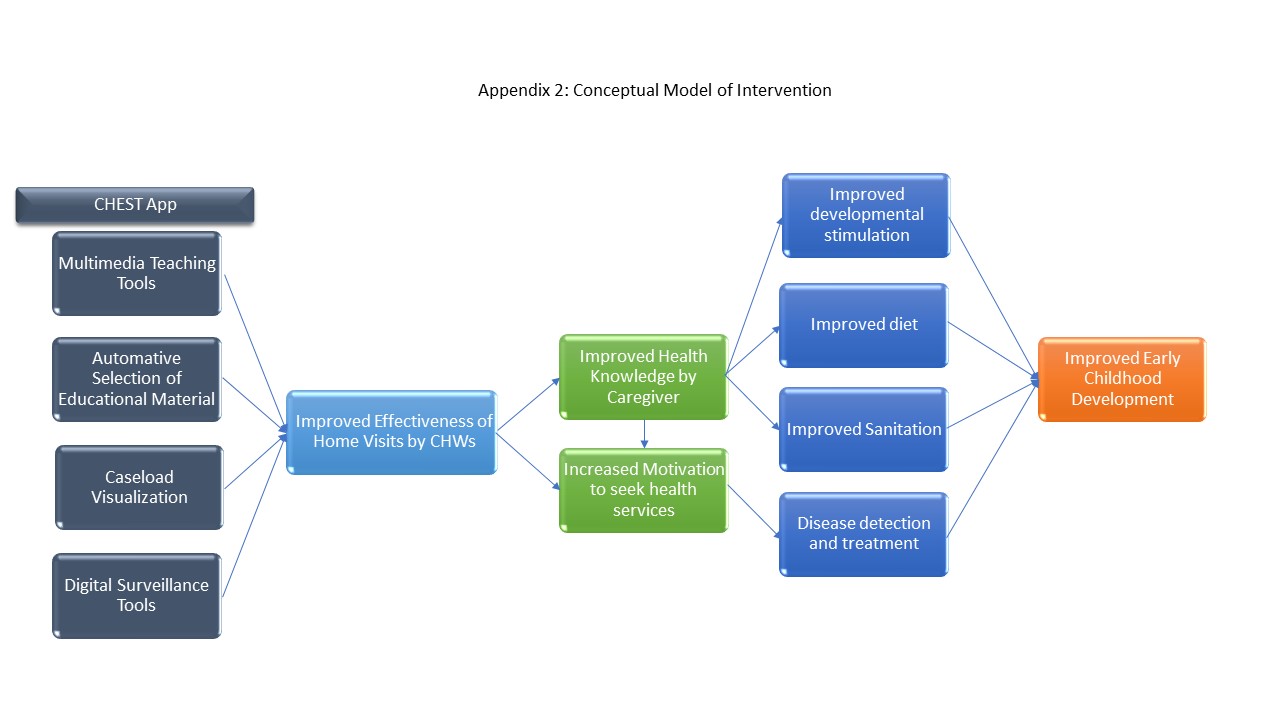

Supplement: S2 Fig — (TIFF) [file pgph.0001118.s002.tiff]

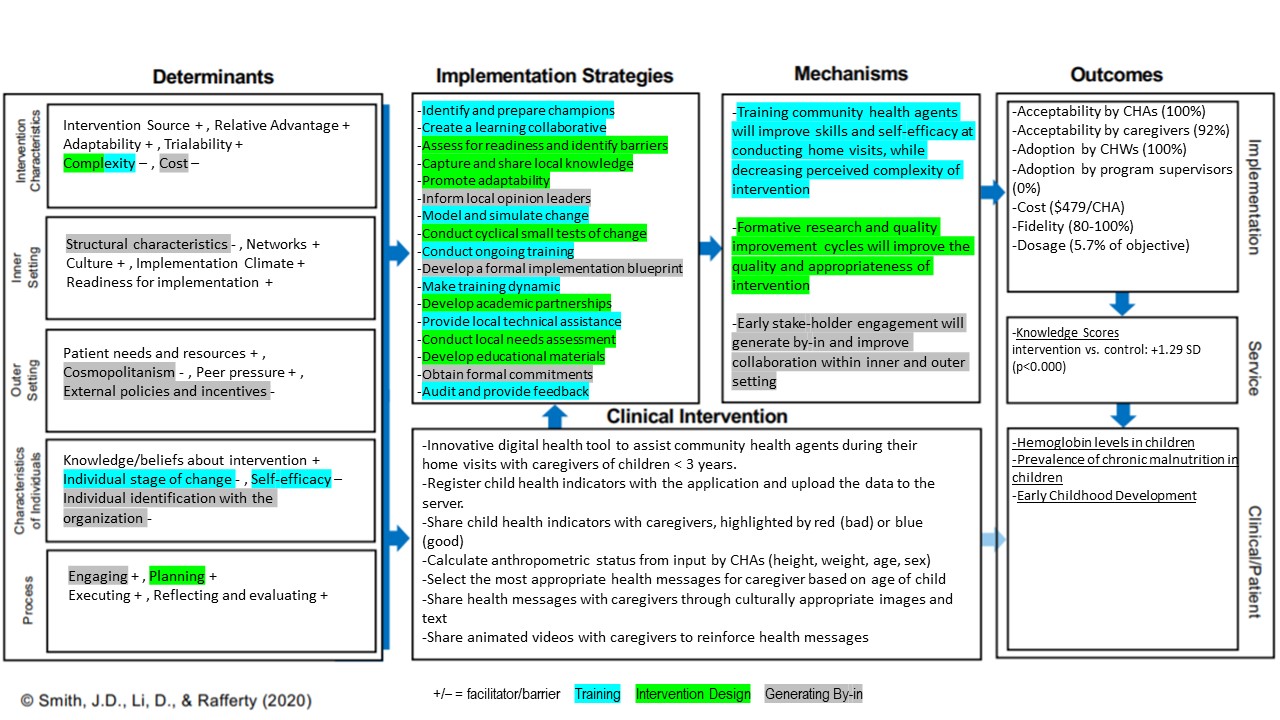

Supplement: S3 Fig — (TIFF) [file pgph.0001118.s003.tiff]

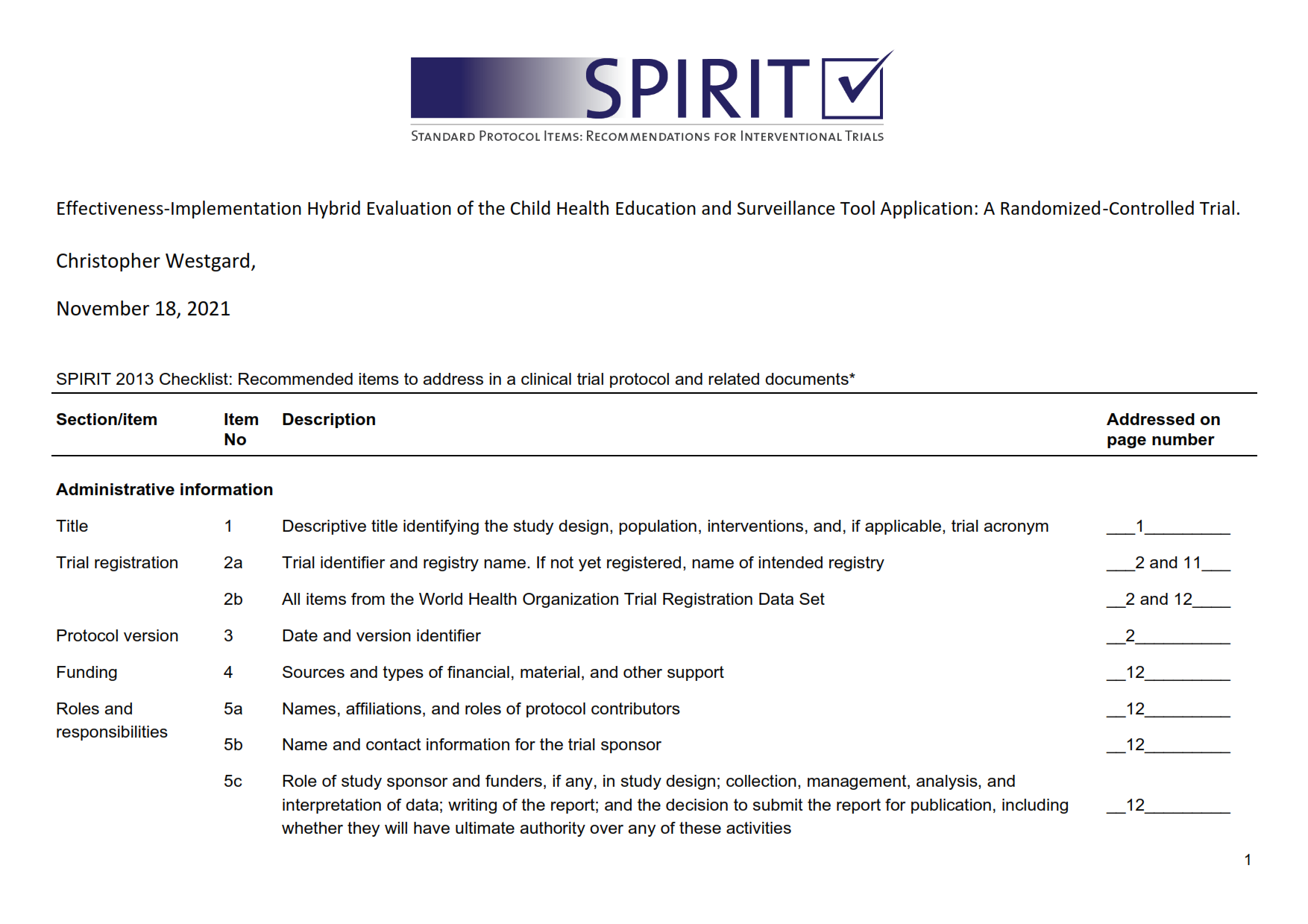

Supplement: S4 Fig — (TIF) [file pgph.0001118.s004.tif]

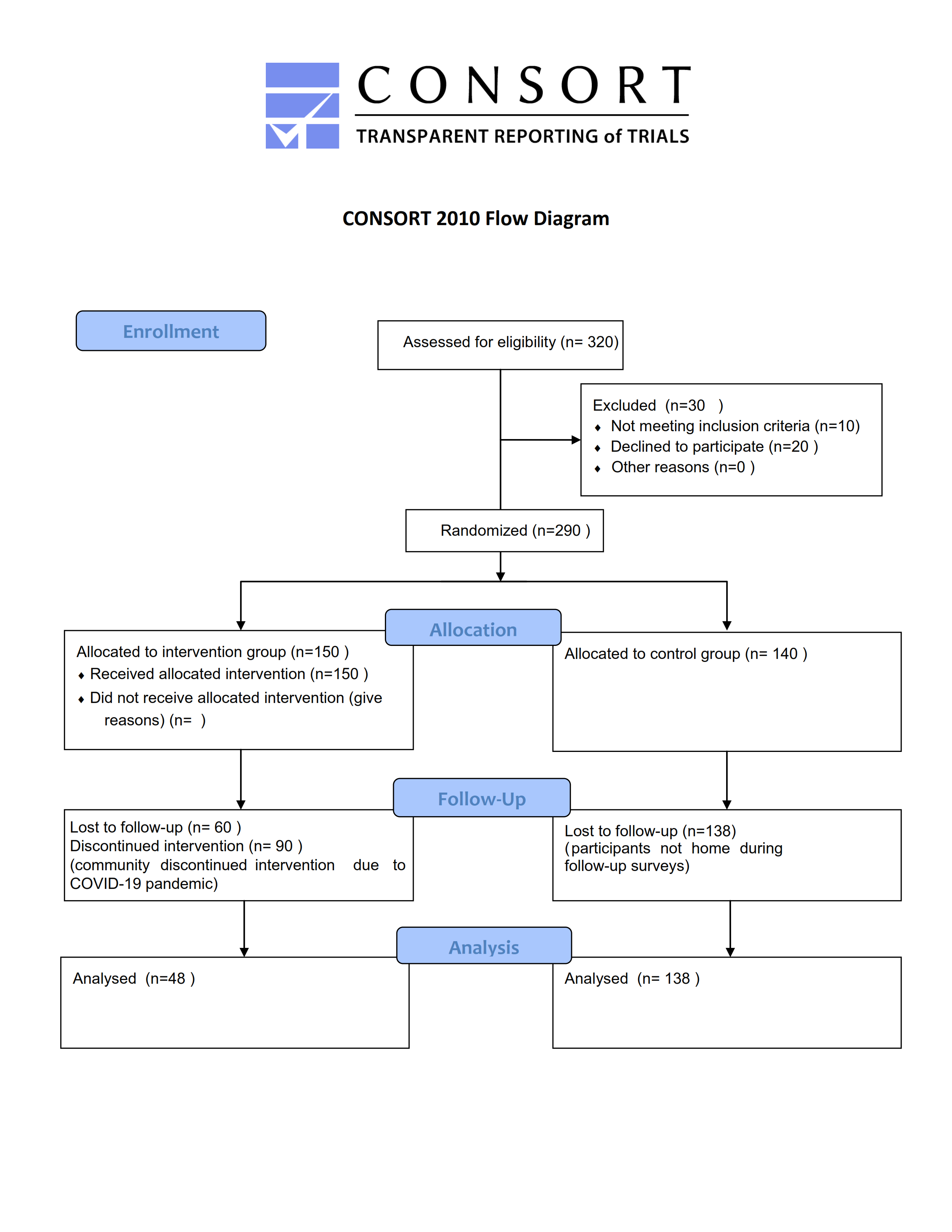

Supplement: S5 Fig — (TIF) [file pgph.0001118.s005.tif]

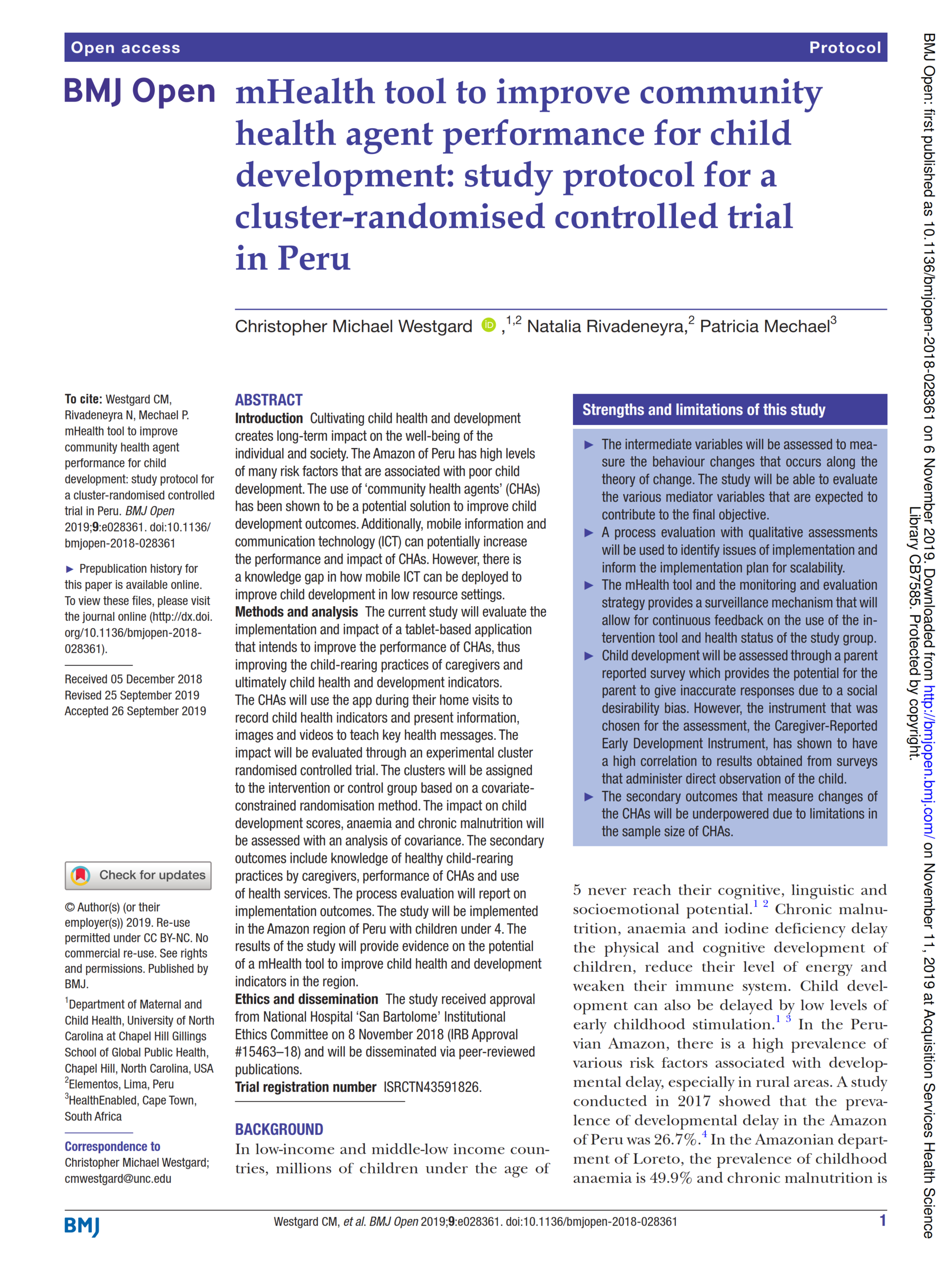

Supplement: S6 Fig — (TIF) [file pgph.0001118.s006.tif]
