# Supplementary material for: An mHealth tool for community health workers to improve caregiver knowledge of child health in the Amazon: An effectiveness-implementation hybrid evaluation
Source: PLOS Glob Public Health. 2022 Sep 22;2(9):e0001118. doi: 10.1371/journal.pgph.0001118 (PMC10021143; doi:10.1371/journal.pgph.0001118)
Supplement: Attachment — Submitted filename: Response to Reviewers. Plos GPH. 7.1.2022.docx [file pgph.0001118.s007.docx]

Thank you to the editor and the reviewers. The suggested edits were extremely helped and resulted in a much better paper. I believe you will find that we have addressed your concerns and greatly improved the manuscript. Thank you all for your time. I hope you will find the final manuscript to be a pleasant read.

Journal Requirements:

1. You indicated that you had ethical approval for your study. In your Methods section, please ensure you have also stated whether you obtained consent from parents or guardians of the minors included in the study or whether the research ethics committee or IRB specifically waived the need for their consent.

The following information was included in the methods section:

All participants provided written consent to participate in the study before receiving any survey or intervention activities. All participants were over the age of 18. Caregivers provided all information related to the children in the study.

2. Please amend your detailed Financial Disclosure statement. This is published with the article. It must therefore be completed in full sentences and contain the exact wording you wish to be published.

a. Please clarify all sources of funding (financial or material support) for your study. List the grants (with grant number) or organizations (with url) that supported your study, including funding received from your institution. 
b. State the initials, alongside each funding source, of each author to receive each grant.
c. State what role the funders took in the study. If the funders had no role in your study, please state: “The funders had no role in study design, data collection and analysis, decision to publish, or preparation of the manuscript.”

The following statement was included in the Financial Disclosure statement:

Project undertaken with the ﬁnancial support of a grant from Saving Brains (#1809-18532), Grand Challenges Canada and the Government of Canada, through Global Aﬀairs Canada (GAC). All authors were supported from the Saving Brains grant. The funders had no role in study design, data collection and analysis, decision to publish, or preparation of the manuscript.

3. Please ensure that Funding Information matches with the Financial Disclosure Statement.

Confirmed

4. Please provide separate figure files in .tif or .eps format and removed from the manuscript file.

For more information about figure files please see our guidelines:

<https://journals.plos.org/globalpublichealth/s/figures> 
<https://journals.plos.org/globalpublichealth/s/figures#loc-file-requirements>

All figure files were converted to .tiff. There are no figures in the manuscript file.

5. Please note that your Data Availability Statement is currently missing direct link to access each database. If your manuscript is accepted for publication, you will be asked to provide these details on a very short timeline. We therefore suggest that you provide this information now, though we will not hold up the peer review process if you are unable.

A Direct link to the database was included in the manuscript, in the section: Availability of Data and Material

https://figshare.com/articles/dataset/Implementation_Research_for_the_Evaluation_of_the_Child_Health_Education_and_Surveillance_Tool_Application/13681423

**Reviewer #1:**

• The first paragraph is very vague and provides no idea about what this manuscript is about.

Thank you for your feedback. The first paragraph was revised to be clearer and more directed towards what the paper is about.

• The authors discuss about CHWs role in conducting health promotion and education. However, it is unclear if these practices are geared toward a certain goal, or generic in nature.

Great point! I added clarification to the sentence.

• The authors state “ICT and mHealth technology have also been shown to be effective at improving health behavior” – unclear about what this behavior is – nutrition, physical activity, routine management, self-care, quality of life – there can be several different areas.

The sentenced was revised to clarify the specific impact of the ICTs.

• In lines 69-70, authors report: “The current study reports on the implementation and intermediary effects of an mHealth innovation to improve CHW performance in the Amazon of Peru.” Unclear presentation of information regarding what the performance is about. What is the outcome of interest?

The sentenced was revised to provider a clearer indication of the primary outcome of the study. The aims of the study and primary outcome is further described in the last paragraph of the background section.

• The allures to use of implementation strategies. Discussing what the outcome is and why knowledge or evidence about this area is limited will strengthen the background section. If this is a broad area, then providing a description what it entails is required.

The background section was revised to provide a better description of the outcomes of interests and limitations of the current state of the field.

• The paper does not really follow a proper methodology reporting. This makes it difficult to gather information. It would be helpful to have a section about design, participants, intervention, instrument, procedure, analysis. The current draft is a mishmash of all of these sections in various combination.

The methodology section was revised and re-organized to include more subcategories and to better group related information.

• The authors refer to a previously published paper for details about methodology. However, some details in this paper are required to understand the results and discussion sections.

More information about the study design was added to methodology.

• The authors didn’t really provide any detail about the CHEST App. As a reader, I am not sure what kind of information was included. The authors report that a video of the app and the animated videos can be found online. However, for the purposes of this manuscript, some details about the content of this app are required to understand what the intervention is about. Without this information, the intervention is only “an app”.

The following information was added to the background section:

The application selects the health messages and animated videos to present during the home visit based on the age of the child, it calculates anthropometric outcomes based on heigh, weight, and age of the child, it provides a caseload screen to display the health status of the child and next scheduled home visit, and it uploads the data collected during the home visit to a server.

• The results are reported in percentages. Not knowing what the total n is makes it difficult to understand what “13%” or “3%” is.

The sample size for each study group was added to the tables and to the data presented in the results section.

• The authors provided p-values for comparative data. However, the authors have not reported if the data within each group were normally distributed allowing them to use parametric statistic.

Great point! The knowledge scores were normally distributed within each group. A sentence clarifying that was added to the results section.

• How were missing data handled?

There were no missing data. We did not need to describe the procedure for missing data.

• Also, for ANOVA results, the authors reported df1 as 2. However, they only reported two treatment levels – intervention and control. This would make df1 = 1. More clarity needed.

Another great point! Thank you. The error was changed.

• The manuscript will benefit from a thorough edit for English language and grammar.

The entire document was revised by both authors. The primary author, a native English speaker, did the final editing.

**Reviewer #2:** The authors examine implementation of an mHealth tool designed to support CHWs in delivering home-based nurturing care services for caregivers and children in rural Peru. The topic is important, as a growing body of evidence suggests that implementation factors are key in determining the effectiveness of CHW home visit interventions that aim to improve child development in low resource settings. The analytic approach is straightforward, and the manuscript is clearly written. I have several comments:

1. The role of CHW supervision has been shown to be very important in determining the effectiveness of home visit interventions in improving child development outcomes. While the mHealth tool is framed as an aid for content delivery, it would be good to better understand its role in the supervision structure. Is there a function in the tool that is meant to strengthen supervision? Is there a supervisor who regularly checks whether CHWs are using the tool? The role of the “program coordinator” within the supervision structure is not clear.

We added information about the supervisors’ use of the App. I understand that more information about the supervisor system would be helpful, but if we add that level of detail of the CHW program the manuscript could become unwieldy. We refer to the previous publication on the CHW program and intervention to provide that level of detail.

The supervisors of the CHWs use the App to monitor the frequency of home visits and the health status of the children. They also assist the CHWs to upload the data from the tablets and use the data for program related decision making. We added information about the supervisors in the Discuss section.

2. The dosage outcome deserves more attention. A key challenge in scaling home visit interventions for child development is ensuring regular home visits occur, which relates in part to the supervision issue mentioned above, as well as the overall way that the CHW workforce is managed and financed (as evidenced by two municipalities in the study area cancelling their CHW programs entirely). That the number of home visits did not reach the intended goal is consistent with experience in other settings. In general, CHWs are often asked to do too much and skipping visits to the homes of ostensibly healthy children is a common way to manage workload. Do the authors have evidence that the reason for the shortfall was due to COVID-19 and not due to other more common factors that could undermine the potential scale-up of the tool? Is there in a function in the tool designed to support achieving the intended dosage, e.g., providing supervisors with an easy check on the number of home visits a CHW conducts? In general, the success of the tool during the home visits that do occur is an important finding, but if a large percentage of home visits do not occur it is difficult to conclude that the tool is an effective and scalable intervention.

The is an extremely important issue. The study was not actively involved with ensuring that the correct doses of home visits was achieved. The study was intended to show that the impact of CHW programs can be enhanced by the use of the tool, independent of enhancing the other aspects of the program (recruitment, supervision, incentives, etc.) Unfortunately, the tool falls short of solving many of the (most) challenging issues of CHW programs. The App was developed with the intention of making supervisory activities easier for the program coordinators (dates of last and next visit displayed on the screen), however, the effectiveness of those functions was not measured. I added a sentence in the background section to further describe the functions of the App.

To your point, there were CHW program cancellations, even before the CHEST App was implemented. I added a sentence clarifying this in the section, *Adoption by CHWs.* Several communities cancelled/paused their CHW program even before the intervention was implemented. We are confident that the drop in doses in the intervention community was due to COVID. The research team was monitoring data obtained with the CHEST App before COVID hit. The CHW coordinators said that they are pausing home visits due to COVID. However, we are unable to know if they would have continued with the expected doses in the absence of the pandemic. Our conclusion is that, if a CHW program is functioning, it will function better with the App. I believe that the findings can translate to effectiveness at scale, however, a large-scale, uninterrupted study is needed to conclude that with full confidence. I added a information in the final paragraph of the discussion section to clarify that.

3. Part of the pathway to impact of CHW home visits on child development outcomes could be through direct interactions between CHWs and children themselves. Are there any aspects of the tool that are targeted at children (and any corresponding data on related implementation factors) or is the tool meant entirely to facilitate information sharing between the CHW and caregiver?

The tool was designed to facilitate the interaction between the CHW and caregiver, only. The content of the App is not meaningful for the targeted children of the study (0-3 years old). I am confident that the content of the App/educational influences spilled over to other family members, especially older siblings. However, we did not have a way to assess that indirect influence on the children, nor the outcomes on other family members. The larger impact evaluation will assess the effect of the intervention on early childhood development. The study needs a larger sample and more time to detect changes at that level.

4. I suggest a slight reframing of the first paragraph to acknowledge that poverty is the root cause of many of the adverse experiences that are detrimental to child development in settings such as this, even if the more proximal causes include “unhealthy practices in the household”. Poverty severely constrains caregivers’ ability to adopt healthy practices irrespective of knowledge.

I agree. I added the following information to the second sentence,

*Although poverty can constrain caregivers’ ability to adopt certain healthy practices in the household, a large portion of ailments can be alleviated by …*

I did not go as far to say that poverty is the root/primary cause. Positive deviant studies in the same communities showed me that other families with the same amount of resources could achieve good health and development. Although I believe that availability of resources is at least equally important to knowledge and attitude, I did not want to quantify one as a greater influence than the other.

**Reviewer #3:**

Overall, the authors did an excellent job in developing the manuscript. Authors report on evaluating an mHealth system (App) implemented to improve CHWs performance in the Amazon of Peru. The manuscript significantly contributes to literature on mHealth innovations in improving healthcare. However, I believe a few items should be addressed before this article is ready for publication. Of particular note, a few grammar issues such as long sentences and grammar errors need to be addressed. Authors should carefully read through the manuscript and correct long sentences that may make it hard for laypersons to read, understand, and appreciate the paper. Authors should also carefully format and structure the manuscript to meet Plos Global Public Health's publication style.

Excellent point. We have revised the entire document and took extra care to ensure there are no run-on sentences.

A few additional comments/suggestions that I have are as follows:

1. The study's main aim is not well stated in the background of the abstract and the manuscript's main text. The sample size is not stated in the abstract of the study.

The sample size was added to the abstract (intervention N=48 and control N=138).

The study aim was added at the end of the background section. The following was added:

The current study aims to evaluate the implementation of the CHEST App intervention and assess the impact of the intervention on the intermediary outcome, knowledge of healthy practices by caregivers. The evaluation will help to identify opportunities to improve implementation strategies during scale-up and replication in other contexts and determine if the intervention is creating the change in the population that is expected (increase knowledge).

2. The methodology of the manuscript should be well described and structured to clearly highlight the significant components of the methodology of a manuscript. The method of analysis is not well described in the methodology section of the main manuscript. Authors should clearly describe in detail the methods of analysis.

The methodology section was significantly revised to better highlight the various methods used to evaluate the program.

3. Line 345 “affective” should be changed to “effective.”

Changed. Thank you.

4. The discussion section of the manuscript section should be well described. Authors should clearly describe how similar or different their findings are from literature. It should describe the social and policy implications of the study findings.

The discussion section was revised to provide more context about the findings and refer to other studies that found similar or conflicting results.
