# Supplementary material for: An mHealth tool for community health workers to improve caregiver knowledge of child health in the Amazon: An effectiveness-implementation hybrid evaluation
Source: PLOS Glob Public Health. 2022 Sep 22;2(9):e0001118. doi: 10.1371/journal.pgph.0001118 (PMC10021143; doi:10.1371/journal.pgph.0001118)
Supplement: Attachment — Submitted filename: Response to Reviewers_v2_PlosGPH_08182022.docx [file pgph.0001118.s008.docx]

Journal Requirements:

1. "Appendix 6. Trial Protocol.tif" file(s) is over our file size limit of 10MB. Please reduce the file size to no more than 10MB. For further help on compressing figures visit: <https://journals.plos.org/globalpublichealth/s/figures>

Appendix 6 was compressed to be below the size limit.

2. Please review your reference list to ensure that it is complete and correct. If you have cited papers that have been retracted, please include the rationale for doing so in the manuscript text, or remove these references and replace them with relevant current references. Any changes to the reference list should be mentioned in the rebuttal letter that accompanies your revised manuscript. If you need to cite a retracted article, indicate the article’s retracted status in the References list and also include a citation and full reference for the retraction notice.

Dear Editor, I cannot find a retracted paper in the bibliography. I conducted a reference check with the software, scite, to identify any references with “editorial concerns”, such as retractions, corrections, or heavily contested research. The reference check found 0 references with editorial concern. I also checked each reference, one-by-one to identify any issues with the reference. I identified two references that needed to be updated with more complete reference to government websites and databases. I updated reference #5 and #57. Will you please point me to any other reference that is creating concern? I will be happy to address the reference.

Additional Editor Comments (if provided):

I understand that the reviewers' comments were attended to, but I would recommend that some attention is given the additional comments from reviewer 3 to help further enhance the quality of the manuscript.

**Comments to the Author**

6. Review Comments to the Author

Please use the space provided to explain your answers to the questions above. You may also include additional comments for the author, including concerns about dual publication, research ethics, or publication ethics. (Please upload your review as an attachment if it exceeds 20,000 characters)

Reviewer #2: (No Response)

Reviewer #3: The authors have failed to respond to most of the critical issues raised by reviewers in the first review concerning the background, methodology, and the results and discussion sections of the manuscript. The significance of this manuscript in enhancing global knowledge of the contribution of mHealth to improving child health cannot be overemphasized. Therefore, authors should carefully revisit the reviewer comments from the first review process to enhance the quality and readability of the paper. Below are some few additional comments:

-Overall, the manuscript is not well formatted. There are issues with paragraphing and paragraph spacing.

The manuscript was reviewed and edited. Following the final revision, the manuscript was reviewed by a faculty member at UNC-Chapel Hill to ensure proper reporting, formatting, and content. The manuscript was then edited by the authors until the faculty member approved the manuscript. I believe the manuscript will now satisfy the expectations of Reviewer #3.

-The background section is still vague and unclear. It should be clearly written and well structured to demonstrate a clear overview of the study. The main purpose of the study should also be stated clearly in the background. For instance, lines 67-68 and lines 73-77 tries to highlight the purpose of the study. However, it is not very clearly stated.

The background section was re-written and reformatted. Additional information was added to provide more background research to support the rational and justification of the study.

The objective of the study was re-written and further clarified at the end of the background section.

-Line 79-108 can be moved to the methods section where the study intervention was described.

The description of the intervention (line 79-108) was moved to the methods section.

-The methodology section should be structured to follow a proper research methodology reporting format. Authors should describe each method section clearly to enhance understanding and readability.

The methods section was re-written, with a special focus on formatting. The sub-sections of the methods section were re-titled and reviewed to enhance understanding and readability.
